# RIPK1 mutations causing infantile-onset IBD with inflammatory and fistulizing features

**DOI:** 10.3389/fimmu.2022.1041315

**Published:** 2022-11-18

**Authors:** Mutaz Sultan, Mohammad Adawi, Nitzan Kol, Blake McCourt, Ihda Adawi, Liran Baram, Noa Tal, Lael Werner, Atar Lev, Scott B. Snapper, Ortal Barel, Liza Konnikova, Raz Somech, Dror S. Shouval

**Affiliations:** ^1^ Department of Pediatrics, Faculty of Medicine, Makassed Hospital, Al-Quds University, Jerusalem, Palestine; ^2^ The Genomic Unit, Sheba Cancer Research Center, Sheba Medical Center, Ramat Gan, Israel; ^3^ Wohl Institute of Translational Medicine, Sheba Medical Center, Ramat Gan, Israel; ^4^ Sackler Faculty of Medicine, Tel Aviv University, Tel Aviv, Israel; ^5^ Department of Pediatrics, Yale Medical School, New Haven, CT, United States; ^6^ Department of Obstetrics, Gynecology and Reproductive Sciences, Human and Translational Immunology, Yale Medical School, New Haven, CT, United States; ^7^ Institute of Gastroenterology, Nutrition and Liver Diseases, Schneider Children’s Medical Center of Israel, Petah Tiqwa, Israel; ^8^ Pediatric Immunology Service, Edmond and Lily Safra Children’s Hospital, Sheba Medical Center, Ramat Gan, Israel; ^9^ Pediatric Department Ward A, Edmond and Lily Safra Children’s Hospital, Sheba Medical Center, Ramat Gan, Israel; ^10^ Jeffrey Modell Foundation Center, Edmond and Lily Safra Children’s Hospital, Sheba Medical Center, Ramat Gan, Israel; ^11^ Division of Gastroenterology, Hepatology and Nutrition, Boston Children’s Hospital, Boston, MA, United States; ^12^ Department of Medicine, Harvard Medical School, Boston, MA, United States

**Keywords:** IBD, RIPK1, monogenic, VEOIBD, inflammasome, Crohn’s disease

## Abstract

**Purpose:**

Receptor-interacting serine/threonine-protein kinase 1 *(*RIPK1) is an important regulator of necroptosis and inflammatory responses. We present the clinical features, genetic analysis and immune work-up of two patients with infantile-onset inflammatory bowel disease (IBD) resulting from *RIPK1 *mutations.

**Methods:**

Whole exome and Sanger sequencing was performed in two IBD patients. Mass cytometry time of flight (CyTOF) was conducted for in-depth immunophenotyping on one of the patient’s peripheral blood mononuclear cells, and compared to control subjects and patients with Crohn’s disease.

**Results:**

The patients presented with severe colitis and perianal fistulas in the first months of life, without severe/atypical infections. Genetic studies identified pathogenic genetic variants in *RIPK1* (Patient 1, A c.1934C>T missense mutation in Exon 11; Patient 2, c.580G>A missense mutation residing in Exon 4). Protein modeling demonstrated that the mutation in Patient 1 displaces a water molecule, potentially disrupting the local environment, and the mutation in Patient 2 may lead to disruption of the packing and conformation of the kinase domain. Immunofluorescence RIPK1 staining in rectal biopsies demonstrated no expression for Patient 1 and minimal expression for Patient 2, compared to controls and patients with active Crohn’s disease. Using CyTOF unbiased clustering analysis, we identified peripheral immune dysregulation in one of these patients, characterized by an increase in IFNγ CD8^+^ T cells along with a decrease in monocytes, dendritic cells and B cells. Moreover, RIPK1-deficient patient’s immune cells exhibited decreased IL-6 production in response to lipopolysaccharide (LPS) across multiple cell types including T cells, B cells and innate immune cells.

**Conclusions:**

Mutations in *RIPK1* should be considered in very young patients presenting with colitis and perianal fistulas. Given RIPK1’s role in inflammasome activation, but also in epithelial cells, it is unclear whether IL1 blockade or allogeneic hematopoietic stem cell transplantation can suppress or cure the hyper-inflammatory response in these patients. Additional studies in humans are required to better define the role of RIPK1 in regulating intestinal immune responses, and how treatment can be optimized for patients with RIPK1 deficiency.

## Introduction

Significant progress has been made in the last 15 years in understanding the role of genetics in the pathogenesis of inflammatory bowel disease (IBD), including Crohn’s disease (CD) and ulcerative colitis (UC) (1). More than 240 single nucleotide polymorphisms, typically with a relatively high mean allelic frequency (MAF) in the general population, have been identified as conferring risk for CD, UC or both (2). On the other hand, rare deleterious variants directly causing intestinal inflammation were identified using advanced sequencing technologies (3, 4). To date, nearly a 100 distinct monogenic disorders associated with IBD were characterized, resulting from deleterious and rare mutations in genes important for immune responses, epithelial cell function, or both (5, 6). Consequently, in many of these diseases intestinal inflammation is accompanied by an immunodeficiency state, manifesting as recurrent or atypical infections. Identification of a monogenic disorder can have a marked impact on care provided for these patients through the possibility of providing targeted therapies, as we and others have shown before with anakinra for patients with IL10 receptor mutations (7) and abatacept for LPS Responsive Beige-Like Anchor Protein (LRBA) deficiency (8).

Mutations in Receptor-interacting serine/threonine-protein kinase 1 (RIPK1) associated with monogenic IBD were recently reported (9, 10). The RIPK1 pathway along with other kinases, including RIPK3 and mixed-lineage kinase domain-like pseudokinase (MLKL) (11) is important for necroptosis, a regulated necrotic cell death mechanism. We present the clinical course, genetic analysis and immune work-up of two patients from unrelated families who presented with severe infantile-onset IBD and were found to harbor deleterious mutations in *RIPK1*.

## Methods

### Whole exome sequencing

The study was approved by the IRB committee at Sheba Medical Center and Rabin Medical Center. Informed written consent was obtained from all individual participants included in the study, including RIPK1-deficient patients, their parents, control subjects and patients with active CD (see below). Whole exome sequencing (WES) was performed for the two index patients using an Agilent v5 Sureselect capture kit and Illumina 2500 sequencing technology. Paired end reads (2X100 bp) were obtained, processed and mapped to the genome. The average sequencing depth of the target region is 92X with 95.63% of bases reached at least 10X coverage. We used the BWA mem algorithm (version 0.7.12) (12) for alignment of the sequence reads to the human reference genome (hg19). The HaplotypeCaller algorithm of GATK version 3.4 was applied for variant calling, as recommended in the best practice pipeline (13). KGG-seq v.08 was used for annotation of identified variants (14) and in house scripts were applied for filtering based on family pedigree and local dataset of variants detected in previous sequencing projects. Likely pathogenicity was assessed if the variant was truncating (splicing or non-sense) or missense; in-frame indels were considered if they were predicted to be pathogenic by online prediction tools including PolyPhen-2, SIFT, CADD and MutationAssessor.

### Immune work-up

Cell surface markers of peripheral blood mononuclear cells (PBMCs) were determined by immunofluorescent staining using flow cytometry (Navios, Beckman Coulter, Brea, CA, US) with antibodies purchased from Beckman Coulter. T cell receptor excision circles (TREC) analysis was performed using DNA extracted from the patient’s PBMCs. The amount of signal joint TREC copies per DNA content was determined by real-time quantitative PCR as previously described (15).

### Sanger sequencing

Exons 4 and 11 of *RIPK1* gene were amplified and sequenced by the Sanger method. Briefly, polymerase chain reaction (PCR) amplification was performed using three sets of the following primers:

exon 4-Fw: CAGAATTTCATGTGAACGTTTCCT

exon 4-Rw: GGCTAAGTCCTCACAAGCAGAA

exon 11-Fw: CTGCCAGTGCATCAACAGCTA

exon 11 Rw: CCCATTCTCCAGCTATGAAGTACA

The PCR reaction took place in a 25-μL volume containing 50 ng of DNA, 10 ng of each primer, 1.5 mM dNTPs, in 1.5 mM MgCl2, PCR buffer, with 1.2 units of Taq polymerase (Bio-Line, London, UK). After an initial denaturation of 5 min at 95°C, 30 cycles were performed (94°C for 30 s, 60°C for 30 s, and 72°C for 30 s), followed by a final extension of 10 min at 72°C. PCR amplicons were sequenced in both directions using a commercial sequencing service [Hy Laboratories, Ltd. (Hylabs) Park Tamar Rehovot, Israel].

### Immunofluorescence staining

We obtained formalin-fixed, paraffin-embedded (FFPE) rectal mucosa specimens from the two RIPK1-deficient patients. As controls we used samples from two control subjects with normal colonoscopies, without a history of IBD (Control 1, 16-years-old male evalulated for bloody stools; Control 2, 14-years-old female with recurrent diarrhea), and two patients with CD (Patient 1, 15-years-old male with active ileo-colonic disease; Patient 2, 10-years-old female with active colonic disease). Rectal FFPE specimens were cut into 5 μm-thick sequential sections, that were then dewaxed in xylene and rehydrated stepwise in descending ethanol series followed by antigen retrieval with Antigen Unmasking Solution, Citrate-Based (Vector laboratories, H-3300) at 96°C for 10 min. The slides were then washed with PBS followed by permeabilization and blocking with PBS with 1% BSA, 3%NDS, 0.05% Tween, 0.025% Triton for 30 minutes at room temperature. The sections were then incubated with primary rabbit anti-human RIPK1 monoclonal antibody D94C12 (3493, Cell signaling technology, dilution 1:50), overnight at 4°C, followed by staining with the corresponding secondary antibodies for 1 hour at room temperature and counterstaining with DAPI, which was included in the mounting medium (GBI Labs, E-19-18, Mukilteo, WA). Samples were visualized by confocal microscope (LSM 800, Zeiss, Oberkochen, Germany). All images within each experiment were acquired under the same conditions.

### Protein Modeling

Visualization of the structural consequences of the mutations to RIPK1 was performed using PyMOL (Version 2.4.1) by using the mutagenesis wizard on the indicated experimentally determined structures.

### Mass cytometry (CyTOF) studies

Peripheral blood mononuclear cells (PBMCs) from a RIPK1-deficient patient, both parents, three controls and three patients with active CD were stained with a panel of metal-chylated surface antibodies targeting markers of major immune cell lineages **(** **Supplementary Table 1** **)** per previously published protocol (16). For controls we used PBMCs from 2 females and 1 male aged 14-17 years, with normal endoscopic evaluation and no concern of IBD, while for the CD group we obtained PBMCs from 3 males, aged 11-17 years, one treated with infliximab and two newly-diagnosed treatment-naïve.

The samples were run on Helios2 mass cytometer (Fluidigm, San Francisco, CA, USA). FCS files obtained were analyzed with premium Cytobank software and pre-gated on CD45^+^/viable/single/DNA^+^ events before initiating the analysis. Normalization beads were used and gated out of the analysis. The data was automatically clustered with Pheongraph through cytofkit package in R and visualized with tSNE using all leukocytes (CD45^+^ cells) as input and manually labeled based on markers expressed in the individual clusters. Cluster abundance (% of CD45^+^ viable single events) and fold change over the RIPK1 abundance were computed and plotted for comparison between groups using Prism8 software. Additionally, clusters of similar cellular subtype were combined, and similarly, the ratio of CD4/CD8 cells was calculated.

To identify dysregulation in activation and cytokine production of the immune cells in RIPK1 deficiency, we stimulated PBMCs from Patient 1 and his parents with either lipopolysaccharide (LPS) or phorbol 12-myristate 13-acetate (PMA) and Ionomycin. PBMC’s from patient and parents were thawed and washed with T cell media, then centrifuged at 300g. The supernatant was discarded and pelleted PBMC’s were resuspended in 1ml of T Cell media. GolgiStop and GolgiPlug were then added to each stimulation tube and each sample was stimulated with either LPS 1mg/ml or PMA 0.5ng/ml and Ionomycin 1mg/ml for 4 hours at 37°C. Samples were then centrifuged and washed again in T cell media then prepped for CyTOF where they were stained with heavy metal chelated antibodies (**Supplementary Table 2**) first for surface antigens, then fixed and permeabilized and finally stained with antibodies for intracellular antigens (16). All samples were analyzed as above. In addition, mean expression of various cytokines (mean metal intensity) was calculated in the various populations indicated.

## Results

### Case description

Patient 1 was referred at the age of 16 months for evaluation of IBD. He was born to a consanguineous Muslim family (parents first degree cousins) and presented at the age of 9 months with fever and diarrhea that were initially attributed to amebiasis. Despite metronidazole treatment his condition deteriorated, and he developed perianal abscesses with multiple fistulas. Blood tests were significant for anemia (hemoglobin 6.5 gram/dL), hypoalbuminemia (albumin 1.5 gr/dL) and elevated inflammatory markers (CRP 94 mg/L, normal <5 mg/L). Due to the severity of the perianal disease, the patient underwent double barrel protective ileostomy. Despite this intervention, his condition did not improve. At the age of 16 months the patient appearead cachectic. Weight was 7.4 kg (Z score -3.2) and length 73 cm (Z score -2.8). Abdominal exam showed double barrel ileostomy, with severe inflammation in the surrounding skin, and multiple perianal fistulas with deep ulcerations and perianal fissures. Colonoscopy demonstrated patchy areas of severe colonic ulcerations with pseudopolyps, with normal mucosa in between, while an upper endoscopy was unremarkable. Colonic biopsies revealed chronic active inflammation with focal cryptitis.

Following a course of broad-spectrum antibiotics and nutritional supplementation, the patient was started on metronidazole, mesalamine and azathioprine, and later began adalimumab. Nevertheless, the patient failed to respond to this TNFα antagonist despite adequate drug levels and absence of anti-drug antibodies, thus experiencing primary pharmacodynamic failure, and at the age of 2.5 years developed colo-vesical fistula that required fistulectomy. Follow up colonoscopy showed severe ulcerations at the sigmoid and ascending colon. Anakinra, an IL1 receptor antagonist, was commenced with escalation of the dose up to 3mg/kg daily, though clinical or laboratory responses were not documented. Importantly, the patient did not develop severe or atypical infections until the age of 3 years.

Patient 2 was also born to a consanguineous Muslim family (parents first degree cousins) and presented at the age of 1 month with recurrent fevers, non-bloody diarrhea, oral ulcers, poor weight gain, abdominal distention and arthritis. During infancy he developed recurrent perianal abscesses. At the age of 20 months his weight was 9.7 kg (Z score -1.4) and length 81 cm (Z score -1.1). Physical exam was noted for abdominal distention and hepatosplenomegaly, along with a draining perianal abscess. Blood tests demonstrated anemia (hemoglobin 8.8 g/dL) and mildly elevated inflammatory markers (ESR 29 mm/hour). Blood cultures were positive for Providencia stuartii and Pseudomonas aeruginosa, which were treated with Piperacillin-Tazobactam for two weeks. Colonoscopy showed patchy colitis, predominantly at the right colon, while an upper endoscopy was unremarkable. Pathologic assessment showed patchy chronic active colitis. The patient temporarily responded to mesalamine, metronidazole and azathioprine, with a 3 kg weight gain over 10 months and normalization of inflammatory markers. However, he continued to suffer from recurrent febrile episodes, oral ulcers, diarrhea, arthritis and perianal abscesses.

### Basic immune studies

Immunoglobulin levels, including IgM, IgA and IgG were within normal limits for both patients (**Supplementary Table 3**). Moreover, immunoglobulin E was also normal for Patient 1. In addition, lymphocyte subset analysis was within normal limits for both patients, beside slightly elevated CD8^+^ T cells (**Supplementary Table 3**). Finally, TREC levels for both patients were within normal limits, reflecting intact thymic function.

### Identification of RIPK1 mutation

**Figure 1:**
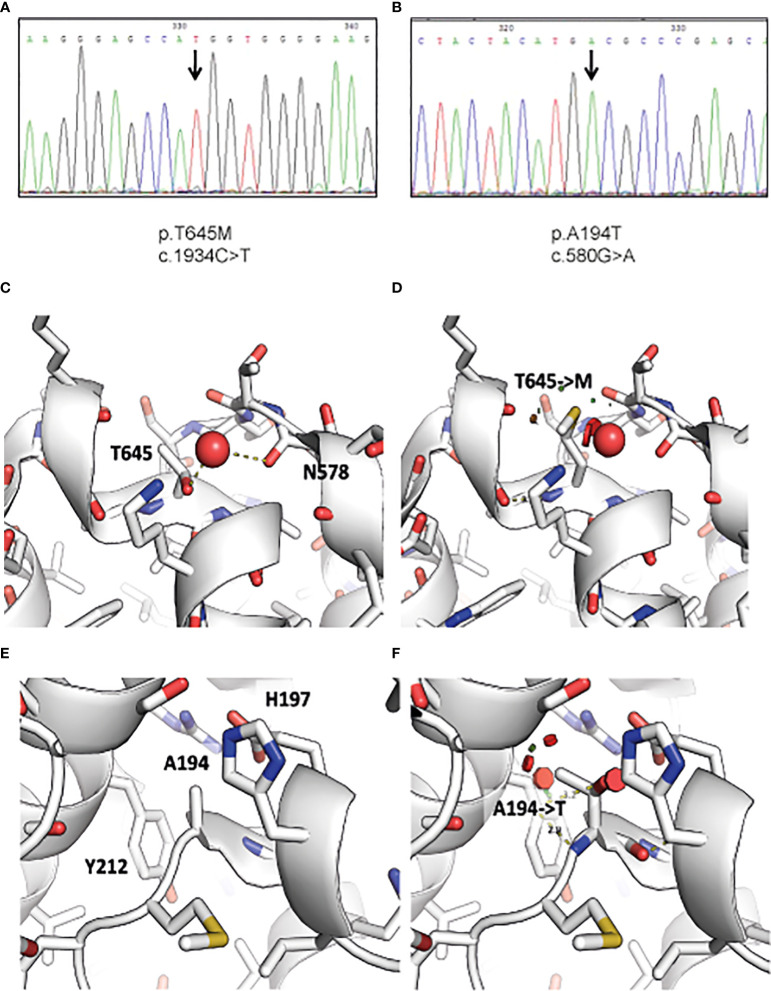
Identification of *RIPK1* mutations in index patients. Figure depicts chromatogram of *RIPK1* gene sequencing at the site of designated mutations for **(A)** Patient 1 and **(B)** Patient 2. Visualization of the structural consequences demonstrates that **(C)** Ala194 is buried in RIPK1 kinase domain C’ lobe (PDB: 4ITJ), and that **(D)** the mutation of Ala194 to Thr introduces clashes (indicated by red disks) with Tyr212 and His197. **(E)** Thr645 is found on the surface of the death domain of RIPK1 and mediates a hydrogen bond to a nearby water molecule (PDB: 6AC5). **(F)** The mutation of Thr645 to Methionine displaces this water molecule and may affect local structure and death domain dimerization.

Following WES analysis, we identified 7,928 and 6,887 homozygous variants for Patients 1 and 2, respectively, that affect protein sequences. These numbers were reduced to 49 and 77 variants, respectively, after filtration for common variants (MAF ≥0.01) in either our local in-house exomes database (n~3500) or external databases such as 1000 Genomes Project (1 KG; https://www.internationalgenome.org/1000-genomes-browsers) or dbSNP 135 database, the NHLBI Exome Sequencing Project (ESP) (http://evs.gs.washington.edu/EVS/) or gnomAD database (https://gnomad.broadinstitute.org/). A c.1934C>T missense mutation residing in Exon 11 was identified in Patient 1, and a c.580G>A *RIPK1* missense mutation residing in Exon 4 was identified in Patient 2 (**Figures 1A, B** **)**. Both mutations were verified using Sanger sequencing. The c.1934C>T mutation has been charactarized previously as a deleterious variant (9). The c.580G>A mutation was assessed as damaging by SIFT and POLYPHEN2 as well as by other tools that predict pathogenicity and had a high CADD score over 29. Moreover the Alanine at position 194 is evolutionarily conserved across most vertebrate species which suggests that it has a role in the RIPK1 function.

To further understand how these variants affect protein function, we visualized RIPK1 structural sequences. RIPK1 is composed of two major structured domains: the kinase domain (aa 17-289) and a death-domain (aa 583-669). There are numerous x-ray structures of the kinase domain (e.g. PDB: 4ITJ) and one crystal structure of the death-domain (PDB: 6AC5). We used these structures to visualize the potential consequence of the mutations we identified.

Thr645 mediates a hydrogen bond to a structured water molecule, that is also bound by neighboring backbone carbonyl of Asn578 (**Figure 1C**). A mutation to Methionine (**Figure 1D**), as identified in Patient 1, would have to displace this water molecule, potentially disrupting the local environment. Moreover, it was shown that even conservative mutations at the death domain (K599R) are sufficient to impair its function (17). In addition, Ala194 is buried in the C’ lobe of the kinase domain (**Figure 1E**). Its mutation to Thr, as identified in Patient 2, may cause significant clashes and as such disrupt the packing and conformation of the kinase domain, which may hamper its activity (**Figure 1F**).

**Figure 2:**
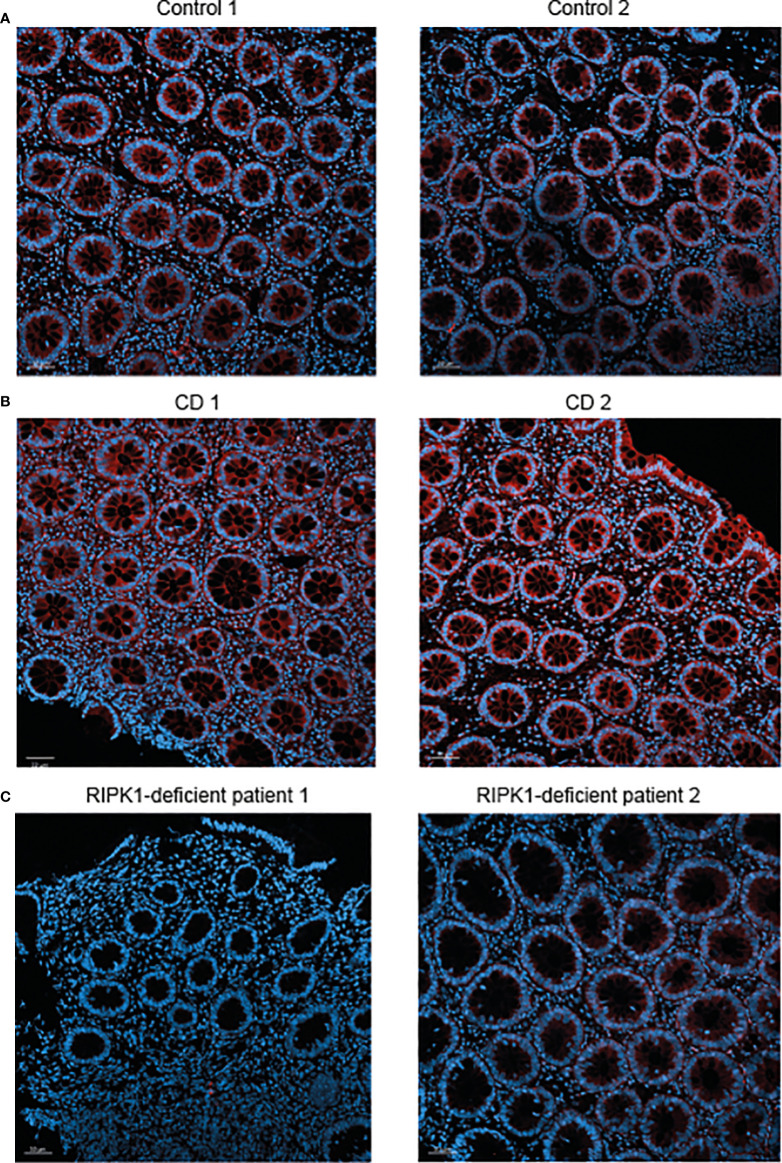
Reduced RIPK1 expression in rectal samples of RIPK1-deficient patients. Figure depicts RIPK1 Immunofluorescence microscopy staining of rectal FFPE sections from **(A)** two control subjects, **(B)** two patients with active Crohn’s disease and **(C)** the two studied patients with RIPK1 deficiency. Sections were stained with anti-RIPK1 antibody (red) and counterstained with DAPI (blue). Magnification 20× (scale bar = 50 µm).

Finally, we performed immunofluorescence staining for RIPK1 in rectal biopsies from the two patients presented above with RIPK1 deficiency. Results were compared to staining in two non-IBD subjects (evaluated for abdominal pain and diarrhea, with normal macroscopic and histologic colonoscopy) and two patients with active CD. In comparison to control subjects, expression of RIPK1 in patients with active CD was increased in rectal tissue (**Figures 2A, B** **)**. Among the two index patients, there was no RIPK1 expression for Patient 1 and minimal expression for Patient 2 (**Figure 2C**).

### Abnormal cytokine production in RIPK1-deficient patient

**Figure 3:**
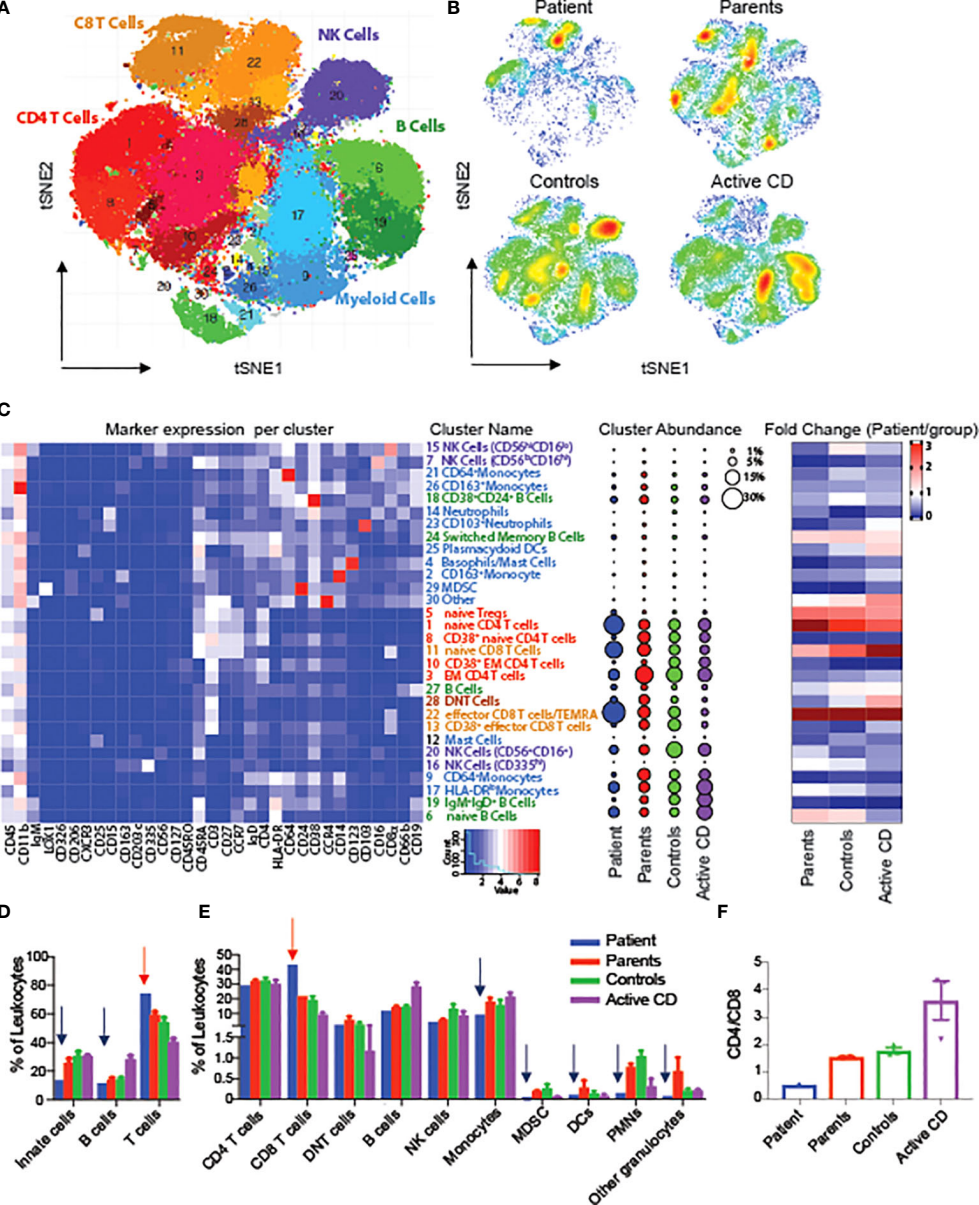
Alterations in circulating leukocytes in a patient with RIPK1 deficiency. tSNE of Phenograph analysis of PBMCs as a **(A)** conglomerate of all samples with individual cellular population shown or as **(B)** density plots of the individual groups. **(C)** Heatmap of the markers expressed in each cluster on the left-hand-side, cluster abundance as a percent of all leukocytes in the middle and fold change of each cluster for each group shown as compared to the RIPK1-deficient Patient 1. **(D, E)** Clusters combined by the immune populations indicated. **(F)** CD4 to Cd8 ratio. Blue arrow-relative reduction and red arrow-relative increase in the RIPK1 patient compared to all other groups.

To evaluate changes in the architecture and function of peripheral immune system in one of the RIPK1-deficient patients, we performed mass cytometry (CyTOF) using a panel of 36 antibody markers, and compared the results to both maternal and paternal blood samples, non-IBD subjects (controls) and CD patients, An unbiased clustering algorithm (Phenograph) was performed on multiple immune populations. To begin, we clustered on all immune cells (CD45^+^) and were able to identify 28 unique populations (**Figures 3A–C**). When comparing abundances of major immune subsets, we identified an increased abundance of T cells, particularly CD8 effector population, in the RIPK1-deficient patient, compared to controls, along with a decrease in all other cell types including monocytes, dendritic cells and B cells (**Figures 3C–E**). Interestingly, we also observed a reversal of the CD4^+^ to CD8^+^ ratio compared to all other samples (**Figure 3F**).

**Figure 4:**
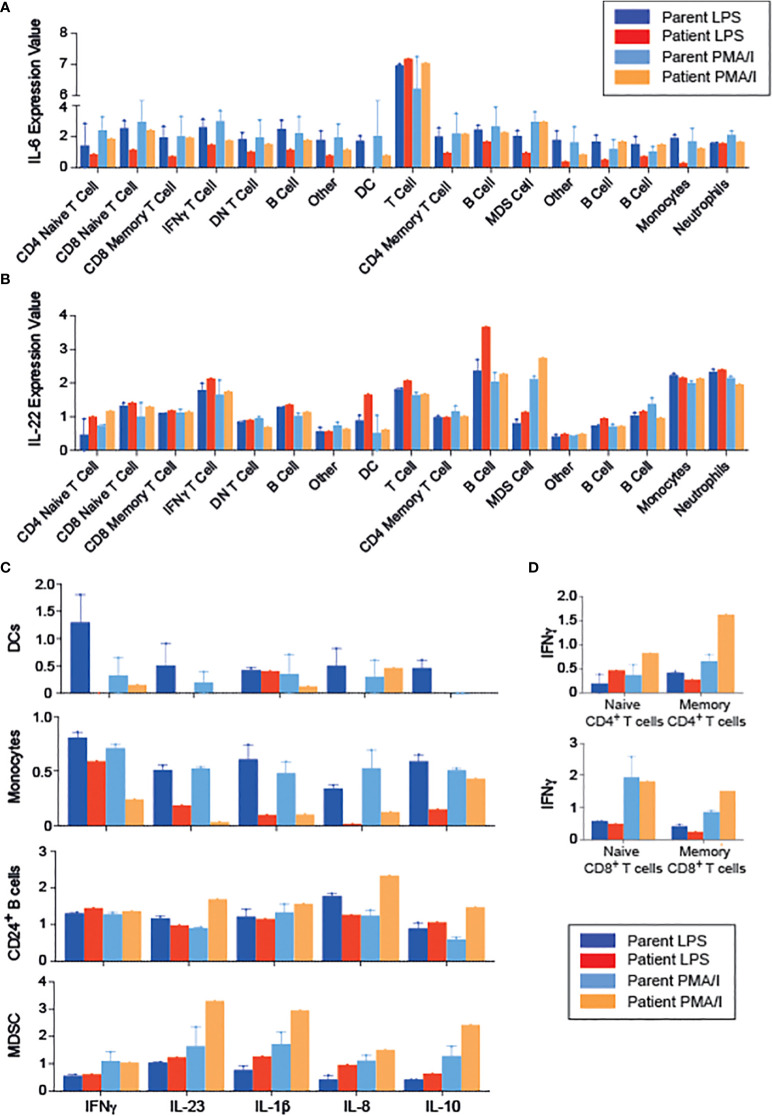
Changes in the cytokine profiles of PBMCs in a patient with RIPK1 deficiency. **(A)** IL-6 and **(B)** IL-22 expression in the various populations indicated obtained from phonograph analysis of stimulated PBMCS from RIPK1-deficienct Patient 1 vs. controls, in response to LPS or PMA/I stimulation. **(C)** Cytokine production by the immune populations indicated. **(D)** IFNγ production with stimulation by LPS or PMA/I in T cells. DC, dendritic cells; MDSC, myeloid-derived suppressor cells.

To gain further insight into the functional effects of RIPK1 deficiency, we examined cytokine production by PBMCs following stimulation with either LPS or PMA/Ionomycin (PMA/I), using CyTOF, and clustering on all leukocytes (CD45^+^ cells, **Supplemental Figures 1A–C**). We were able to identify 17 unique populations, again demonstrating a CD8 predominance in the patient (**Supplemental Figures 1A, B)**. RIPK1-deficient patient’s immune cells exhibited decreased IL-6 production in response to LPS, but not to PMA-I stimulation, across multiple cell types including T cells, B cells and innate immune cells (**Figure 4A**). In addition, we observed an increase in IL-22 production across the majority of immune cells, particularly in response to LPS stimulation condition (**Figure 4B**).

Focusing on the individual cell populations, monocytes and dendritic cell’s ability to produce inflammatory cytokines was drastically reduced in the patient, consistent with documented reduced NFkB activity (18) (**Figure 4C**). On the other hand, patient’s T cells produced higher amounts of IFNγ than healthy controls upon PMA/I stimulation (**Figure 4D**). Interestingly, when analyzing immune populations with inhibitory potential, including myeloid derived suppressor cells (MDSC) and CD24^+^ B cells (containing the putative regulatory B cell populations), we demonstrate higher production of inflammatory cytokines (IL-23, IL1β, and IL-8), but also IL-10, compared with cells obtained from the parents (**Figure 4C**), suggesting a possible dysfunction of these cells.

## Discussion

With the expanding use of next generation sequencing platforms in patients with unique IBD phenotypes, the list of monogenic disorders associated with intestinal inflammation is steadily growing, and many of them present with an increased susceptibility to severe and/or atypical infections. RIPK1 is a central regulator of apoptosis, inflammation, and necroptosis, and therefore, it is not surprising that patients that lack RIPK1-dependent signaling develop a severe multi-systemic disease with inflammatory phenotypes and infectious complications in some of them. To date, 14 patients with deleterious *RIPK1* mutations have been reported in the literature (9, 10, 19, 20). All patients developed colitis, mostly in the first months of life, and in all but two patients perianal inflammation (abscesses or fistulas) was evident (9, 10). Chronic upper gastrointestinal tract inflammation was also reported as a phenoyte of RIPK1 deficiency (19). Additional clinical features included susceptibility to severe viral and bacterial infections, arthritis, skin lesions, recurrent fever and oral ulcers. Differences in clinical phenotype may be attributed to the functional consequences of the *RIPK1* mutation and whether it abrogates protein expression. This disorder should not be confused with a different autosomal dominant autoinflammatory disease caused by heterozygote mutations in *RIPK1*, leading to its abnormal cleavage by caspase-8, and manifesting as recurrent fever, lymphadenopathy and hepatosplenomegaly (21).

The patients we present here manifested with severe colitis and perianal inflammation in the first months of life, but without recurrent severe infections. Nevertheless, due to their young age it is possible that susceptibility to specific pathogens as well as additional inflammatory manifestations will develop later in life. Both patients harbored variants that are predicted to be deleterious to protein function based on genetic and protein modeling. We did identify minimal expression of RIPK1 protein in rectal samples from Patient 2, but this does not necessarily imply normal function, especially given the high pathogenicity score of the genetic variant. Interestingly, expression of RIPK1 was increased among the two patients with CD, compared to controls. Additional larger-scale studies are required to define whether this observation is consistent in states of active IBD, and whether it implies enhanced inflammasome activation in the inflamed tissue.

One of the pathways that has been shown to be upregulated in RIPK1-deficient macrophages is activation of the inflammasome, an intracellular complex that induces the secretion of the pro-inflammatory cytokines IL-1β and IL-18 in response to different stimuli, including LPS (22). Impaired inflammasome activation has been reported in other monogenic disorders associated with intestinal inflammation, including Caspase-8 deficiency (23), mutations in IL-10 receptor (IL10R) (7), mevalonate kinase (MVK) deficiency (24) and in gain-of-function mutations of *NLRC4 (* 25). We have previously shown than anakinra, an IL1 receptor antagonist, was effective in suppressing intestinal inflammation in two patients with deleterious *IL10RA* mutations and history of severe infantile-onset IBD (7), and, similarly, blocking IL-1 is effective in patients with MVK deficiency (26). Over-activation of the inflammasome in patients with *RIPK1* mutations may suggest that IL-1 blocking agents can be effective in suppressing the IBD phenotype, though additional pro-inflammatory cytokines and pathways may also be dysregulated and contribute to the hyper-inflammatory immune response observed in this disorder. A trial of anakinra (IL-1 receptor antagonist) was performed in Patient 1, but the dose provided was only 3mg/kg, much lower than the does we successfully used for the IL10R-deficient patients (10mg/kg) (7).

Our CyTOF data of PBMCs from a single RIPK1-deficient patient highlighted alterations in both innate and adaptive immune cells. The patient displayed increased frequency of naïve CD4^+^ and CD8^+^ T cells, compared to controls as well as patients with active CD, similar to observations by Li and colleagues (9). In addition, we demonstrated decreased IL-6 production by various innate and adaptive immune subsets. This finding is in-line with reports from Cuchet-Lourenco and colleagues who found decreased ERK and NfkB phosphorylations and a reduction in pro-inflammatory cytokines such as IL-6 in RIPK-deficient fibroblasts. In addition, the TLR4-dependent (LPS-induced) signaling was affected more than TLR independent pathways (10). The dysfunction of B cells and MDSC has been reported in IBD patients (27, 28); however, this is the first report, to the best of our knowledge, in RIPK1 deficient patients.

The question whether hematopoietic stem cell transplantation (HSCT) can cure RIPK1 deficiency is unclear at this point. Cuchet-Lourenço and colleagues reported 3 siblings (One was sequenced and found to harbor a *RIPK1* mutation and the others with the presumable same mutation) that underwent HSCT; two of them died within weeks of the procedure due to multi-organ failure or disseminated viral infections. The third patient underwent HSCT at the age of 30 months with resolution of IBD and arthritis (up to 5 years of follow-up), though is still on antibiotics due to chronic and probably irreversible lung disease (10). Mice with a specific deletion of RIPK1 in epithelial cells develop spontaneous lethal intestinal inflammation due to enhanced apoptosis and necroptosis of epithelial cells (29, 30). Moreover, RIPK1-deficient intestinal epithelial cells show altered cell death responses in response to TNFα stimulation (9). Given RIPK1’s roles in governing both immune and epithelial responses, using HSCT to cure these patients should be carefully considered, as it might ameliorate the immunodeficiency phenotype but not intestinal inflammation. This might be similar to Nuclear factor-kappa B essential modulator (NEMO) deficiency due to *IKBKG* mutations, in which HSCT eliminates the increased susceptibility to recurrent/atypical infections, but does not cure the IBD phenotype (31).

In conclusion, mutations in RIPK1 are a new monogenic form of IBD that should be suspected in patients with very early-onset IBD with inflammatory and fistulizing features. Given RIPK1’s involvement in regulating inflammasome function it is plausible that targeting IL-1 and/or IL-18 may be effective, to some extent, in suppressing intestinal inflammation. Nevertheless, murine and human data, as well as *in vitro* studies, suggest that RIPK1 is important for epithelial cell function, and therefore, therapies targeting immune cells may be only partially effective in these patients. Moreover, the ability of HSCT to cure this disorder is still unclear. Additional clinical studies and observations are required to determine the role of RIPK1 in epithelial cells in sustaining mucosal homeostasis.

## Data availability statement

The datasets for this article are not publicly available due to concerns regarding patients' anonymity. Requests to access the datasets should be directed to the corresponding author.

## Ethics statement

The studies involving human participants were reviewed and approved by IRB committee, Sheba Medical Center. Written informed consent to participate in this study was provided by the participants’ legal guardian/next of kin.Written informed consent was obtained from the minor(s)’ legal guardian/next of kin for the publication of any potentially identifiable images or data included in this article.

## Author contributions

MS, MA, IA – data collection, manuscript editing. NK, BM, LW, AL, OB, LK, RS, NT, LB – data analysis, manuscript editing. SS – conceptualizing of study, data analysis, manuscript editing. DS - conceptualizing of study, coordination of study, data analysis, manuscript writing. The study was approved by the IRB committee at Sheba Medical Center and informed consent was obtained from all individual participants included in the study or their parents. All authors contributed to the article and approved the submitted version.

## Funding

This study was funded by the Leona M. and Harry B. Helmsley Charitable Trust.

## Acknowledgments

We woud life to thank Prof. Nir London from the Weizmann Institute of Science for his help with protein modeling.

## Conflict of interest

DS declares speaker’s fees from Abbvie and research grant from Takeda. SS declares the following interests: Scientific advisory board participation for Pfizer, Pandion, Celgene, Lilly, Takeda, Cosmo Pharmaceuticals, Merck, Sonoma Biotherapeutics, and EcoR1. Grant support Pfizer, Amgen Takeda, and Novartis. Consulting for Amgen, Kyverna, Bristol Myers Squibb, Third Rock, 89bio, GentiBio and Apple Tree Life Sciences.

The remaining authors declare that the research was conducted in the absence of any commercial or financial relationships that could be construed as a potential conflict of interest.

## Publisher’s note

All claims expressed in this article are solely those of the authors and do not necessarily represent those of their affiliated organizations, or those of the publisher, the editors and the reviewers. Any product that may be evaluated in this article, or claim that may be made by its manufacturer, is not guaranteed or endorsed by the publisher.
