# Supplementary material for: RIPK1 mutations causing infantile-onset IBD with inflammatory and fistulizing features
Source: Front Immunol. 2022 Nov 18;13:1041315. doi: 10.3389/fimmu.2022.1041315 (PMC9716469; doi:10.3389/fimmu.2022.1041315)
Supplement: Supplementary file 1 [file DataSheet_1.pdf]

Supplemental Figure 1

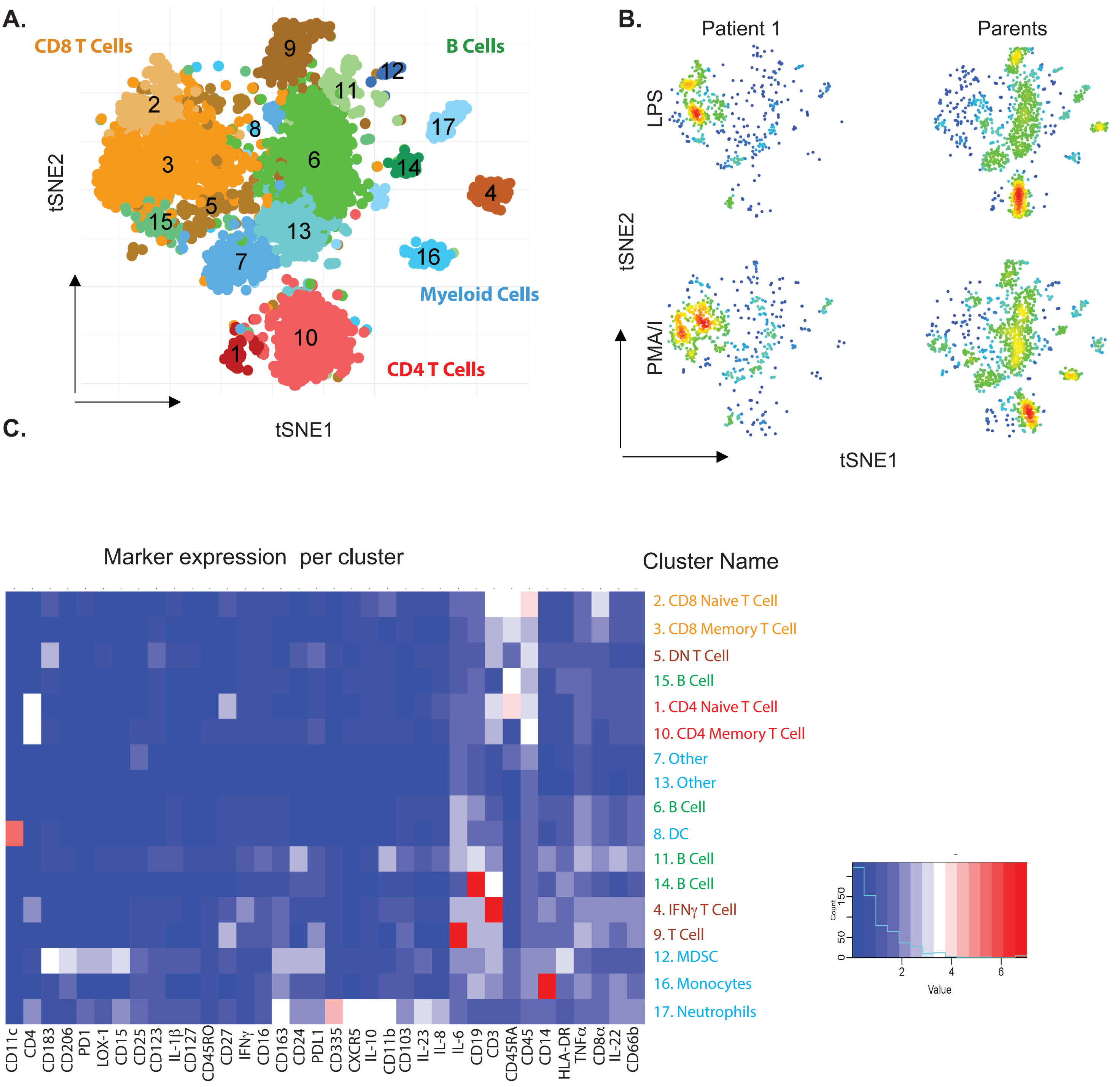

Supplemental Figure 1. Immune dysregulation in RIPK1 deficiency. tSNE of Phenograph analysis of peripheral blood mononuclear cells (PBMCs) stimulated with either LPS or PMA/I as a (**A**) conglomerate of all samples with individual cellular population shown or as (**B**) density plots of the individual groups. (**C**) Heatmap of the markers expressed in each cluster

Metal	Antibody
89	CD45
115	CD11c
141	CD66b
142	CD19
143	HLA-DR
144	CD64
145	CD16
146	CD8α
147	CD45RO
148	CD103
149	CD25
150	CD38
151	CD123
152	CD14
153	CD45RA
154	CD163
155	CD27
156	CCR4
158	CD3
159	CCR7
161	CD203c
162	CD56
163	CD183 (CXCR)
164	CD15
166	CD24
167	LOX-1
168	CD206
169	CD11b
171	CD127
172	IgM
173	CD335
174	CD4
175	IgD
209	CXCR5

Supplementary Table 1. Surface antibody panel used for general phenotyping CyTOF analysis.

Metal	Antibody
89	CD45
115	CD11c
141	CD66b
142	CD19
143	HLA-DR
144	TNFα
145	CD16
146	CD8α
147	CD45RO
148	CD103
149	CD25
150	IL-22
151	CD123
152	CD14
153	CD45RA
154	CD163
155	CD27
156	IL-6
158	CD3
159	CCR7
160	IFNγ
161	IL-23p19
162	IL-1β
163	CD183 (CXCR)
164	CD15
166	CD24
167	LOX-1
168	CD206
169	CD11b
170	IL-8
171	CD127
172	PDL1
173	CD335
174	CD4
175	PD1
176	IL-10

Supplementary Table 2. Antibody panel used for stimulated CyTOF analysis.

Blood test	Patient 1	Patient 2	Normal range
IgG	669	951	700-1600 mg/dL
IgA	270	77	70-500 mg/dL
IgM	105	46	26-100 mg/dL
IgE	69		0-90 mg/dL
TREC	2,287	2,179	>400 copies/0.5 mcg DNA
WBC (cells/mm^3)	21,000	6,790	
Lymphocytes (cells/mm^3)	8,127 (38.7%)	3,530 (52.0%)	
CD3 (cells/mm^3)	6,339 (78.0%)	2,471 (70.0%)	
CD4 (cells /mm^3)	2,601 (32.0%)	741 (21.0%)	436-1394 cells/mm^3
CD8 (cells/mm^3)	3,576 (44.0%)	1,377 (39.0%)	166-882 cells/mm^3
CD20 (cells/mm^3)	650 (8.0%)	812 (23.0%)	50-300 cells/mm^3

Supplementary Table 3: Laboratory work-up of the patients.
